# Mapping and monitoring land use land cover dynamics employing Google Earth Engine and machine learning algorithms on Chattogram, Bangladesh

**DOI:** 10.1016/j.heliyon.2023.e21245

**Published:** 2023-10-24

**Authors:** Jayanta Biswas, Md Abu Jobaer, Salman F. Haque, Md Samiul Islam Shozib, Zamil Ahamed Limon

**Affiliations:** Urban and Rural Planning Discipline, Khulna University, Khulna, 9208, Bangladesh

**Keywords:** Land use land cover (LULC), Urban sustainability, Machine learning, Google Earth Engine, Chattogram

## Abstract

Land use land cover change (LULC) significantly impacts urban sustainability, urban planning, climate change, natural resource management, and biodiversity. The Chattogram Metropolitan Area (CMA) has been going through rapid urbanization, which has impacted the LULC transformation and accelerated the growth of urban sprawl and unplanned development. To map those urban sprawls and natural resources depletion, this study aims to monitor the LULC change using Landsat satellite imagery from 2003 to 2023 in the cloud-based remote sensing platform Google Earth Engine (GEE). LULC has been classified into five distinct classes: waterbody, build-up, bare land, dense vegetation, and cropland, employing four machine learning algorithms (random forest, gradient tree boost, classification & regression tree, and support vector machine) in the GEE platform. The overall accuracy (kappa statistics) and the receiver operating characteristic (ROC) curve have demonstrated satisfactory results. The results indicate that the CART model outperforms other LULC models when considering efficiency and accuracy in the designated study region. The analysis of LULC conversions revealed notable trends, patterns, and magnitudes across all periods: 2003–2013, 2013–2023, and 2003–2023. The expansion of unregulated built-up areas and the decline of croplands emerged as primary concerns. However, there was a positive indication of a significant increase in dense vegetation within the study area over the 20 years.

## 1 Introduction

Human-environment imbalanced interaction drives nature to the brink of hazard, adversely impacting the surrounding ecosystem. Cities are particularly exposed to climate change because of high density, as more than 54 % of the world's population lives in urban areas [1]. Rapid population growth increases the demand for infrastructure development, altering the land cover to a massive extent. Research demonstrates the negative impact of LULC changes on ecosystems and natural assets [2]. Major LULC changes, including urbanization, deforestation, wetland loss, and soil degradation, have a severe impact on civilization, such as air pollution, biodiversity loss, increased deteriorating human health, and extreme weather events [[3], [4], [5], [6]]. This phenomenon is becoming a continuous threat to both current and future generations to meet their needs in a sustainable manner.

Acknowledging this risk, monitoring land-use land-cover (LULC) changes has become critical [7,8]. Learning about LULC change is vital for recognizing spatial and temporal changes in land features and planning for sustainable community development. Agricultural land, flora, forest cover, rivers, canals, ponds, impermeable surfaces, soil, and rock formations are all a part of the Earth's surface [1,9]. Researchers have found that LULC changes can have a wide variety of effects on ecosystem services and products [2].

Responding to these challenges, several methods exist to classify remotely sensed images. The most common procedure is supervised classification (maximum likelihood algorithm). Machine learning algorithms are gaining popularity in assessing LULC classification and seeing the changes over time [10]. Machine learning involves algorithms that enable the computer system to learn the system's behavior based on the input observation and ensure potential classification for non-linear systems. It helps understand the behavior of a system based on input observation. Therefore, a machine learning algorithm is a suitable choice in the classification of remote sensing images, where it is near to impossible to have complete knowledge of the characteristics of the whole study area [11,12]. Moreover, machine learning classifiers are reported to produce higher accuracy even with complex data and a higher number of input features [13,14].

Researchers are shifting towards cloud-based platforms for remote sensing applications as it is more efficient than downloading a large dataset, processing the data manually, and then starting classification. Researchers worldwide are increasingly utilizing Google Earth Engine (GEE) to understand the transitioning patterns of LULC more effectively [[15], [16], [17], [18]]. Data from different satellites (Landsat, Sentinel, MODIS) are gathered in one place on the GEE platform. Moreover, the GEE platform allows researchers to use different machine learning algorithms (Random Forest, 10.13039/100012513CART, Support Vector Machine, and Naive Bayes) for image classification. As machine learning algorithms can be trained with smaller datasets and require less computational resources compared to more advanced algorithms like deep learning [19]. However, deep learning has several drawbacks, including complexity, cost, and the need to wait longer for results. On the other hand, machine learning is straightforward [19,20], this created a huge motivation to use these machine learning algorithms in this study.

Since the combination of GEE and RS usage can handle small to big-scale real change scenarios around the globe, it has been becoming the top choice when researchers working on spatiotemporal quantification of changing dynamics of natural landscapes [[21], [22], [23]].

Many datasets allow investigators to capture natural environments' spatial patterns and the reasons for their alteration based on monitoring historical data of LULC information. Understanding the extent and direction of the shift of LULC changes can help policymakers and planners make appropriate decisions. Massive population movement from Bangladesh's rural and hill tract areas exacerbates the southeastern valley regions. Consequently, this region, formerly celebrated for its high terrain, green cover, and pleasant climate, has seen its metropolitan districts transform into concrete jungles with scant flora cover and a growing urban heat island [24,25]. Studies on assessing the LULC patterns are necessary to bring environmental harmony to this region. Additionally, figuring out the most accurate method to estimate the LULC classification can support future researchers in monitoring transformations of the surrounding environment competently. This research can potentially inform environmental engineers, city planners, and legislators about how to lessen the negative impact of LULC shifts and make a livable environment in an expanding metropolis.

## 2 Study area

**Fig. 1:**
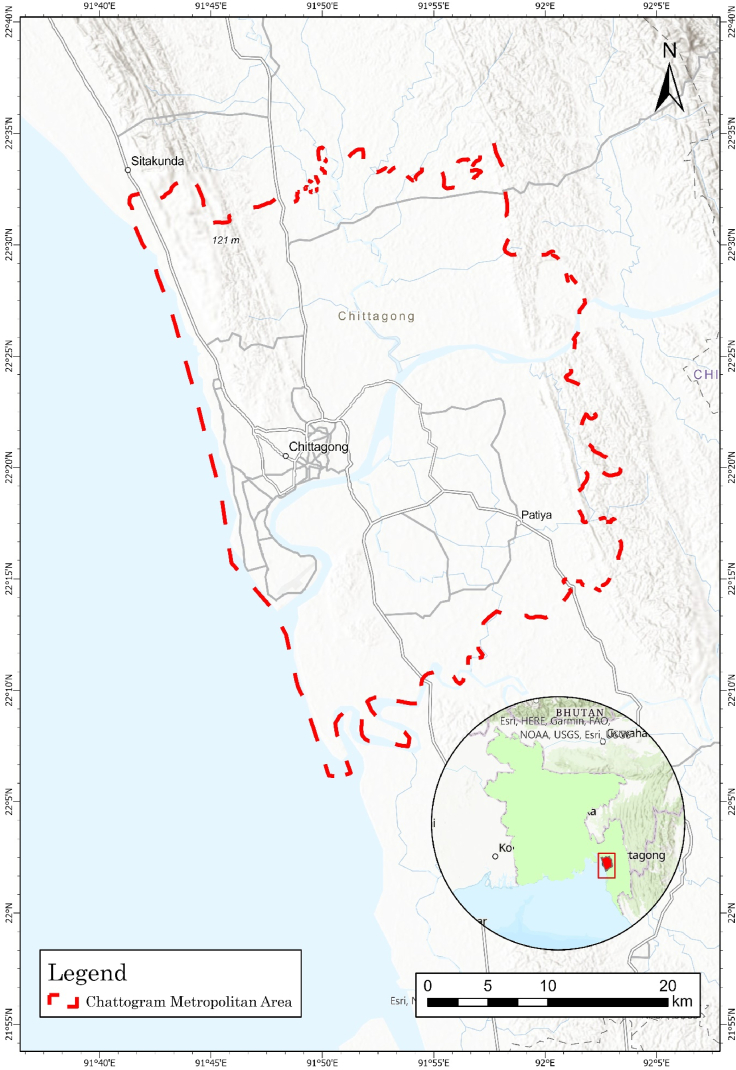
Study area map: Chattogram metropolitan area.

This research considered the Chattogram Metropolitan Area (CMA) as the study area in the southeastern part of Bangladesh. The CMA is the second-largest commercial capital in Bangladesh (Fig. 1) [26]. The Karnaphuli River, the Halda River, the Bay of Bengal, and the Rangamati district all border the CMA to the southwest, northeast, west, and east, respectively [27]. The latitude and longitude of the Chattogram Metropolitan City range from 22.34190000 to 91.81553600, with an approximately 1160 km^2^ area. In addition to being the country's primary entry port, Chattogram is the third busiest international seaport in South Asia [28]. Chattogram climatic conditions fall into tropical monsoon as per Köppen climate classification [29]. The lowest surface elevation of the city is 2 m, and the highest is 117 m. The average summer temperature in the CMA is 22–32° Celsius. In comparison, the average winter temperature ranges from 20 to 26° Celsius, according to data from the Bangladesh Metrological Department.

The Chattogram-Dhaka Highway links the CMA to the nation's capital, Dhaka. Hills and the Bay of Bengal bound the research area. Chattogram City is a significant hub connecting Bangladesh's core tourism destinations. The city draws people from the surrounding countryside and hills, as well as from further afield because it is home to the region's most prominent industrial sector, which provides them with plentiful employment prospects., The enormous influx of migrants is forming rapid changes in land surface usage, such as increasing build-up area, which raises the surface temperature and landslide risk. Furthermore, in recent years, the region's environment, inhabitants' ability to make a living, and agricultural growth have all been negatively impacted by the region's growing urbanization.

## 3 Data and method

### 3.1 Dataset preparation

The 30-m spatial resolution satellite imageries of Landsat-5 (TM) and Landsat-8 (OLI/TIRS) were used for this study. This research considered multi-temporal Landsat satellite images from 2003 to 2023, with each period separated by ten years. This study has been carried out image classification on the Google Earth Engine platform. For classification, only cloud-free images were selected [30], [31,32]. The satellite imageries are directly accessed using a command line code from the GEE platform [30], [[31], [32], [33]]. The image information (Satellite name, acquisition date, name of the sensors, cloud coverage, scene ID, and path/row) is presented in Table 1.

**Table 1:** Imagery information.

Satellite Name	Acquisition Date	Sensor	Cloud Coverage (%)	Scene ID	Path/Row
Landsat 5	2003-11-20	TM	2.00	LT51360442003340BKT00	136/44
Landsat 5	2003-11-20	TM	0.00	LT51360452003324BKT00	136/45
Landsat 8	2013-12-01	OLS_TIRS	0.00	LC81360442013351LGN01	136/44
Landsat 8	2013-12-01	OLS_TIRS	0.00	LC81360452013335LGN01	136/45
Landsat 8	2023-01-11	OLS_TIRS	0.00	LC81360442023011LGN00	136/44
Landsat 8	2023-01-11	OLS_TIRS	0.00	LC81360452023011LGN00	136/45

**Fig. 2:**
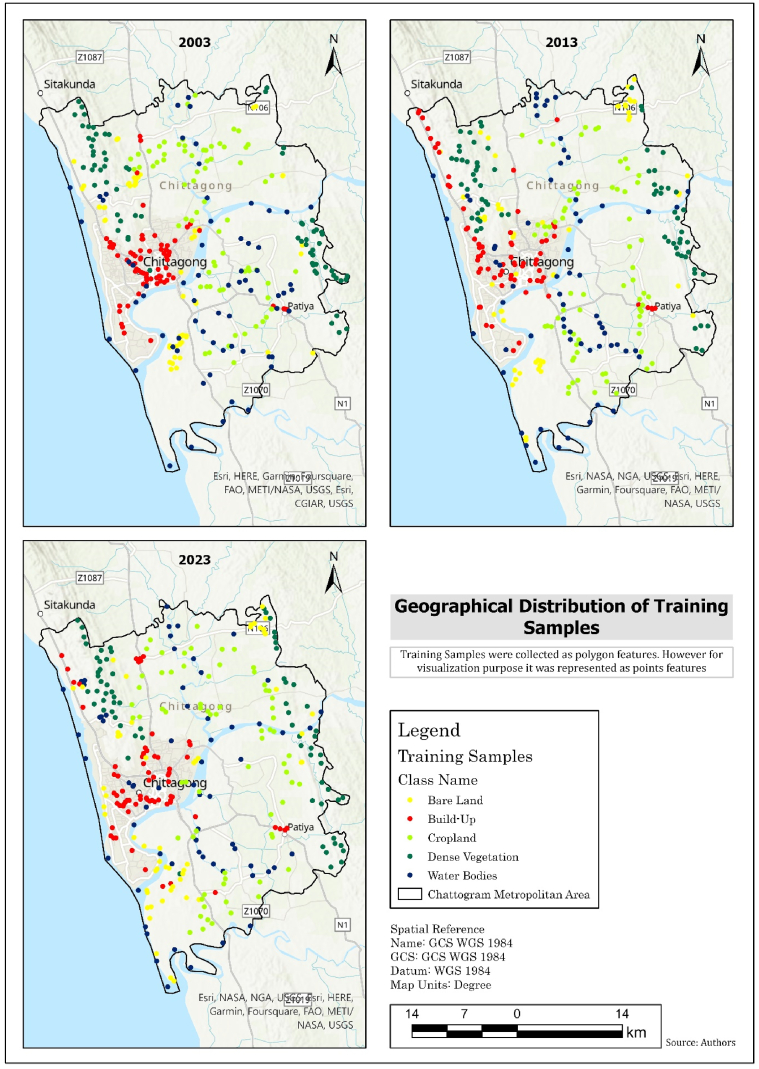
Geographical distribution of training samples.

**Fig. 3:**
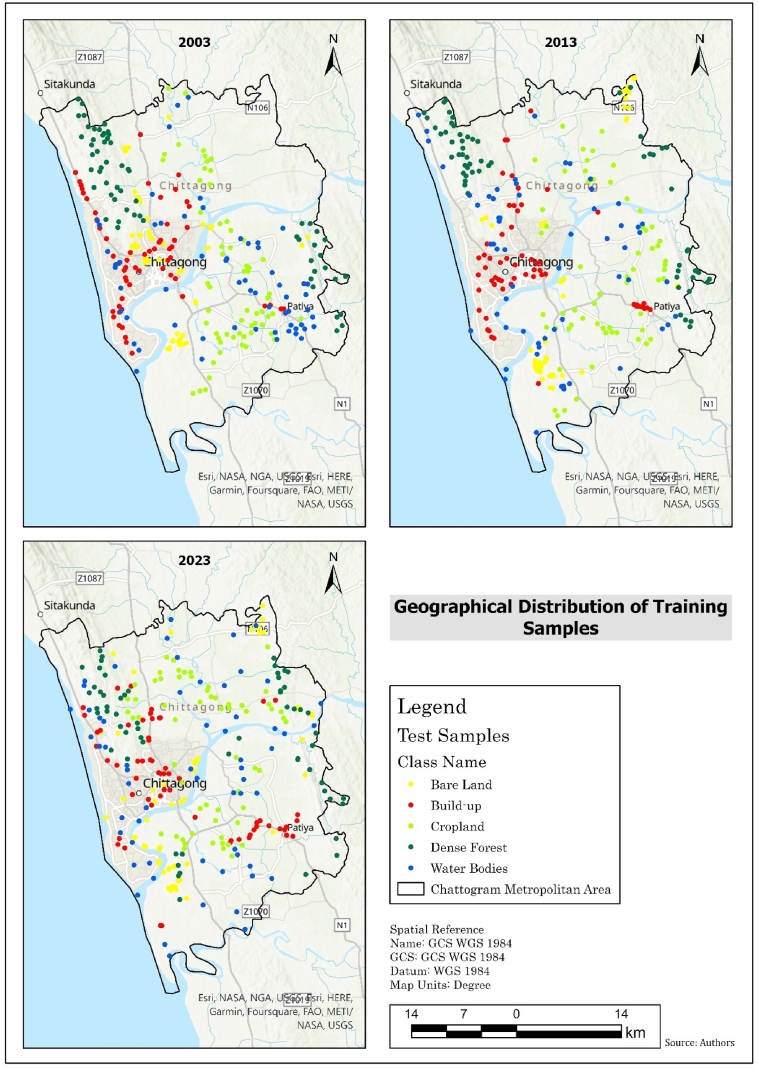
Geographical distribution of test samples.

After calling the Landsat 5 and 8 imageries, the imageries are spatially filtered by the study area's shapefile (CMA Area). Then the spatial filter data is further filtered by a temporal filter. This study has screened data from late November to early January in 10 years intervals from 2003 to 2023. The study tried to retrieve the data within a single season as in Bangladesh winter start in December and ends in February. The dataset has also used another temporal filter from the selected imageries to select only the cloud-free imageries. At the final data processing stage, the cloud-free imageries are sorted date-wise, and the most recent imageries are selected for image composition. The composite image has selected bands 2, 3, 4, 5, 6, 7, and 10 from Landsat 8 and bands 2 to 7 from Landsat 5 for LULC classification. The test and training data set were collected using Google Earth Pro. For training, 330 samples were collected. Table 2 and Fig. 2 represent the class-wise and geographical distribution of training samples. From each class 60 samples in total 300 samples were collected for testing purpose (see Fig. 3).

**Table 2:** Class-wise training sample distribution and pixel count.

Class Name	2003		2013		2023	
	Sample Size	Pixel Count	Sample Size	Pixel Count	Sample Size	Pixel Count
Build-up	70	230	70	226	70	489
Bare Land	55	149	55	497	55	243
Cropland	70	230	70	313	70	941
Dense Vegetation	70	986	70	494	70	361
Water Bodies	65	648	65	1060	65	804

### 3.2 Methods

**Fig. 4:**
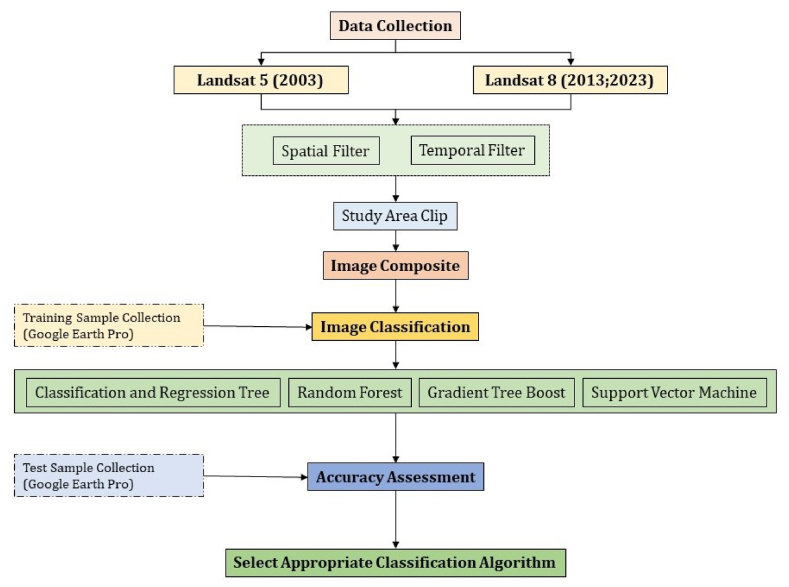
Methodological flow diagram for image classification.

The key steps, including image preprocessing, finding the best classifier, image classification, and accuracy assessment, have been done in the GEE platform. The flowchart (Fig. 4) shows the methodology followed in this study to achieve the objectives. QGIS and ArcGIS Pro are used for classified area calculation, change detection, and map preparation. Microsoft Power BI and Excel are used to produced charts and graphs. The main LULC class structure for the CMA area is shown in Table 3. The study used five main classes (e.g., water bodies, build-up, bare-land, dense vegetation, and cropland) that represent the overall land cover classification using the different classifiers (see Section 3.2.1) on the GEE using Landsat 5 and Landsat 8 imageries from 2003 to 2023 (see Table 4).

**Table 3:** Classification categories.

Class Name	Class Descriptions
Water Bodies	Lakes, bays, oceans, rivers, ponds, and estuaries.
Build-up	Residential, commercial, industrial, roads and transportation, and utilities.
Bare Land	Beaches, an open field without vegetation, transitional areas, and mixed barren land.
Dense Vegetation	Deciduous forest area, mixed forest, residential forest, evergreen forest.
Cropland	Grass, agricultural land, grass.

**Table 4:** Overall accuracy for different classifiers.

	Overall Accuracy			
Algorithm/Year	GTB	RF	CART	SVM
2023	0.890	0.883	0.860	0.707
2013	0.873	0.887	0.863	0.740
2003	0.843	0.887	0.883	0.788

#### 3.2.1 Classification methods

This study employed four machine learning algorithms for land use land cover classification. We collected training samples from each class each year to conduct a supervised classification using pixels. For each LULC course throughout the study, Google Street View, Google Earth Pro, and Google Satellite Image were mined for training samples. As part of the GEE framework, we employed the Support Vector Machine (SVM), Random Forest (RF), Classification and Regression Tree (CART), and Gradient Tree Boost (GTB) classifiers for this investigation. From this investigation, the study tries to find out optimal classification ensemble for the study area.

##### 3.2.1.1 Classification and regression tree (CART)

**Fig. 5:**
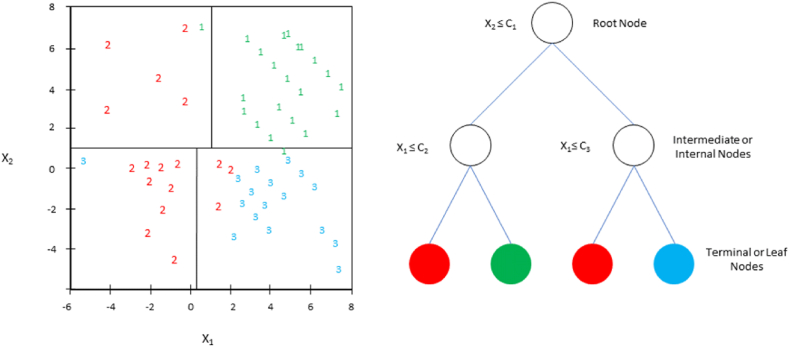
Visualization of classification and regression tree (CART).

CART is one of the simplest binary classifiers, a typical decision tree model developed by Ref. [34] based on the framework of hierarchical decision trees (Fig. 5). The fundamental advantage of simple binary architectures is that classification choices can be understood and interpreted as input-output relations as a white box system [35] (see ).

This classifier is frequently employed in many studies in the remote sensing sector, such as developing MODIS global land cover data [36], mapping flash-flood susceptibility [37], and recognizing black and odorous water bodies [38] leveraging its simplicity. To evaluate the effectiveness of the assessment, CART offers the chance of misclassification at each leaf node. CART outperforms neural networks for lower dimensional data and produces results equivalent to neural networks. On the contrary, CART's performance is decreased due to the complicated tree structures produced by high-dimensional data. Moreover, the sample size picked for each class significantly impacts CART [39].

##### 3.2.1.2 Random forest (RF)

**Fig. 6:**
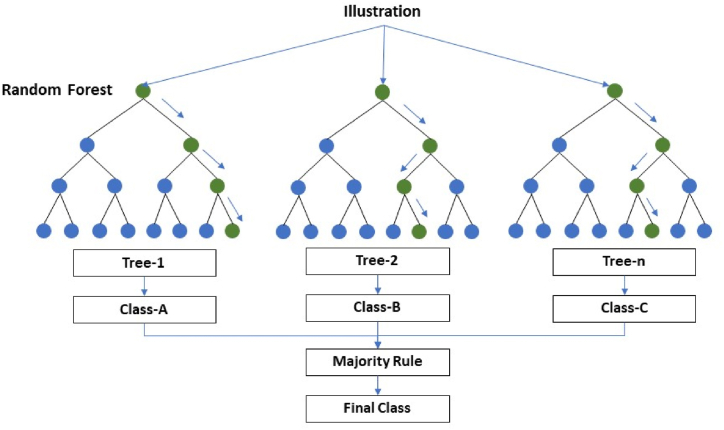
Classification model visual representation.

Tumer and Ghosh (1996) [40] demonstrate that using the combined output from several classifiers to predict an outcome result in extremely high classification accuracy. The ensemble classifier RF, which aggregates the output from various decision trees to select the label for incoming input data according to the majority vote, is built on this principle [41] (see Fig. 6). RF has become more significant due to its resistance to noise and outliers [42]. Additionally, RF outperforms alternative ensemble classifiers that use bagging and boosting, among other ensemble methods [43]. Even applying in a variety of applications, such as the classification of urban landscapes [44], LULC classification using multi-temporal and multi-frequency SAR data [45], urban land use and land cover change analysis [46] and so on.

##### 3.2.1.3 Support vector machine (SVM)

**Fig. 7:**
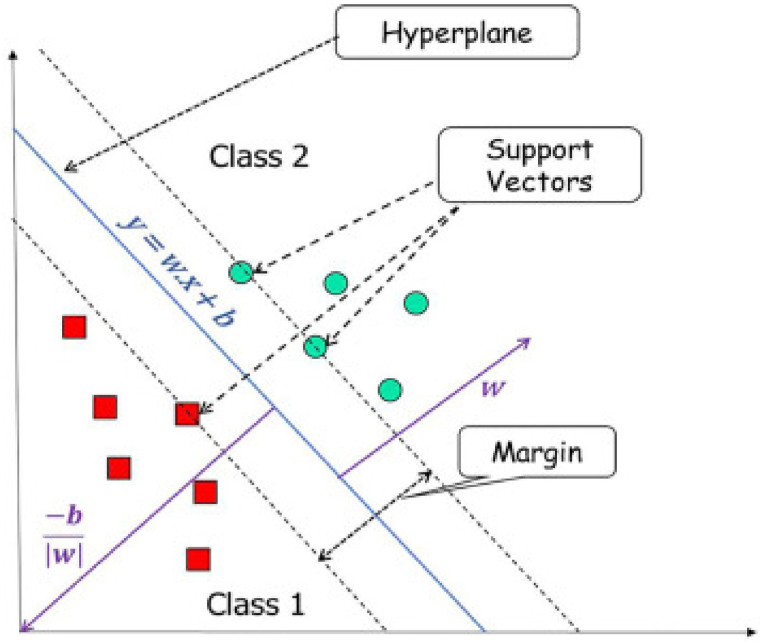
Illustration of support vector machine (Source: [49]).

Support Vector Machine (SVM) is the remote sensing study's primary classifier. SVM earned its reputation as it can accurately classify using lesser training samples [47]. SVM is also a linear binary classifier that follows the concept that training samples close to a class's boundaries will discriminate a class better than other training samples (see Fig. 7). It focuses on finding an optimal hyperplane that separates the input training samples of various classes. It used one-against-all and one-against-one techniques to solve multiclass classification problems.

##### 3.2.1.4 Gradient tree boost (GTB)

**Fig. 8:**
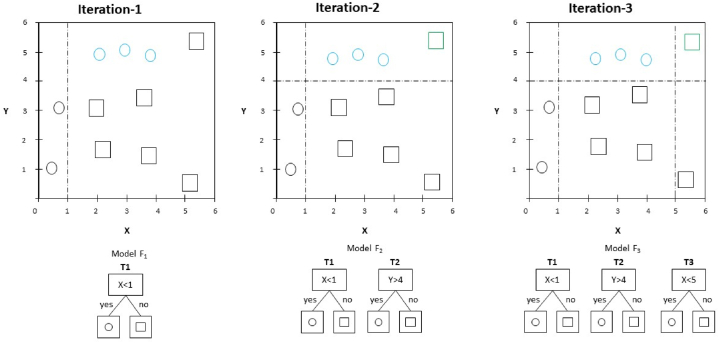
Visual representation gradient tree boosting.

The GTB algorithm uses the iterative combination of weak learner ensembles into more robust ensembles of trees through stepwise minimization of the loss function based on gradient descent optimization to obtain accuracy [48] (see Fig. 8). GTB likes RF and an aggregated group of decision trees (). However, it limits each tree to a weaker prediction model, reducing the decision trees' complexity.

The GTB uses negative gradient loss values to fit the residual of the regression tree at each iteration, making it distinguishable from other ensemble learning methods. It can capture higher-order information, is insensitive to sample data scaling, and can effectively minimize overfitting using a weighting combination technique.

#### 3.2.2 Accuracy assessment

The classification accuracy is affected by classification methods, procedures, time, and space. Several studies found that LULC classification's accuracy varied from a bit to a lot when different LULC classification algorithms were used. The accuracy of different classifiers was used to judge how well they worked. Overall accuracy, which shows the percentage of correctly tested data, is the most common way to measure the accuracy and efficiency of all classifiers. The accuracy evaluation in this study indicates that the outputs of the classifiers used in this case are not the same. The accuracy of a LULC classification depends not only on the classifier but also on where and when it is done (e.g., changes in the surface, air, or light). Randomly, from each category, 300 points were collected for accuracy assessment. The accuracy assessment method was done by making an error matrix for each of the five time periods. Then, statistical procedures like user accuracy (UA) (Equation (2)), producer accuracy (PA) (Equation (3)), overall accuracy (OA) (Equation (1)), and kappa coefficients (Equation (4)) were used.

(1) $$Overall\;Accuracy=\frac{total\;number\;of\;corrected\;classified\;pixels\;\left(diagonal\right)}{total\;number\;of\;reference\;pixels}\times 100$$

(2) $$User\;Accuracy=\frac{number\;of\;correctly\;classified\;pixels\;in\;each\;class\;\left(diagonal\right)}{total\;number\;of\;refeence\;pixels\;in\;each\;category\;\left(row\;total\right)}\times 100$$

(3) $$Producer\;Accuracy=\frac{number\;of\;correctly\;classified\;pixels\;in\;each\;class\;\left(diagonal\right)}{total\;number\;of\;refeence\;pixels\;in\;each\;category\;\left(column\;total\right)}\times 100$$

(4) $$Kappa\;Coefficient=\frac{total\;number\;of\;sample\times total\;number\;of\;corrected\;sample-\sum \left(col.tot\times row\;tot\right)}{{Total\;number\;of\;sample}^{2}-\sum \left(col.tot\times row\;tot\right)}\times 100$$

Besides the PA, UA, and kappa coefficient, this study also evaluates the Receiver Operating Characteristic (ROC) curve to measure the accuracy of LULC classification based on sensitivity and specificity, and the area under the ROC curve (AUC) is also measured. At various categorization thresholds, the ROC curves showed graphs of true positive rates (TPR) versus false positive rates (FPR). The odds of positive classes were anticipated and used to set the thresholds. ROC curves often show how a classification model trades off its TPRs and FPRs. A model with ROC curves closer to the top-left corner would indicate better performance. The ROC curve is quantified numerically as the area under the curve (AUC), calculated from the two-dimensional area under the ROC curve. A higher AUC value implies better classification performance.

(5) $$TPR=\frac{True\;Positive}{True\;Positive+False\;Negative}$$

(6) $$FPR=\frac{False\;Positive}{Fasle\;Positive+True\;Negative}$$

(7) $$AUC=\int_{0}^{1}TPR\;d\left(FPR\right)$$

## 4 Result and discussion

### 4.1 Accuracy assessment of LULC

This study has used four classification methods to classify the images from 2003 to 2023 of Chattogram Metropolitan Area. The accuracy of each classifier is assessed using four different parameters: user accuracy, producer accuracy, overall accuracy, and kappa coefficients. In this accuracy assessment, we have considered five classified categories: build-up, dense vegetation, cropland, bare land, and waterbody (see Table 3). The overall accuracy of the GTB, RF, and CART exceeds 80 % each year. In contrast, the SVM classifier performs relatively poorly in our case. The SVM classifier producer and user accuracy are lesser than other classifiers. In 2013, the SVM algorithm failed to classify the build-up area. In 2013 and 2023, the values of PA and UA weren't that satisfactory for the SVM classifier for our study area. Other classifiers, PA and UA, are satisfactory, besides failing to classify the build-up area accurately. The PA and UA will be less than 60 % in 2023 (see Table 5). The Kappa statistics for GTB, RF, and CART are satisfactory (see Table 6).

**Table 5:** User and producer accuracy table.

	CART		GTB		RF		SVM	
	(%)							
2003	PA	UA	PA	UA	PA	UA	PA	UA
Waterbody	96.67	96.67	93.33	93.33	96.72	98.33	98.33	98.33
Build up	87.27	80.00	83.33	66.67	80.00	86.67	73.58	65.00
Dense Vegetation	100.00	100.00	93.75	100.00	96.77	100.00	90.16	91.67
Bare Land	86.96	66.67	95.12	65.00	97.37	61.67	95.65	36.67
Cropland	74.68	98.33	66.67	96.67	78.38	96.67	53.40	91.67
**2013**	**PA**	**UA**	**PA**	**UA**	**PA**	**UA**	**PA**	**UA**
Waterbody	90.48	95.00	93.22	91.67	98.21	91.67	96.43	90.00
Build up	88.68	78.33	87.93	85.00	87.72	83.33	88.57	51.67
Dense Vegetation	94.92	93.33	93.65	98.33	92.19	98.33	83.87	86.67
Bare Land	75.81	78.33	80.36	75.00	80.70	76.67	77.08	61.67
Cropland	82.54	86.67	81.25	86.67	84.85	93.33	48.48	80.00
**2023**	**PA**	**UA**	**PA**	**UA**	**PA**	**UA**	**PA**	**UA**
Waterbody	98.31	96.67	98.31	96.67	98.31	96.67	96.72	96.72
Build up	85.11	66.67	91.67	73.33	91.30	70.00	59.57	46.67
Dense Vegetation	90.91	100.00	93.75	100.00	93.75	100.00	65.52	95.00
Bare Land	83.87	86.67	94.34	83.33	96.08	81.67	94.44	56.67
Cropland	72.73	80.00	72.37	91.67	70.00	93.33	48.57	56.67

**Table 6:** Kappa statistics.

	Kappa Statistics			
Algorithm/Year	GTB	RF	CART	SVM
2023	0.862	0.854	0.825	0.633
2013	0.842	0.858	0.829	0.675
2003	0.804	0.858	0.854	0.734

**Fig. 9:**
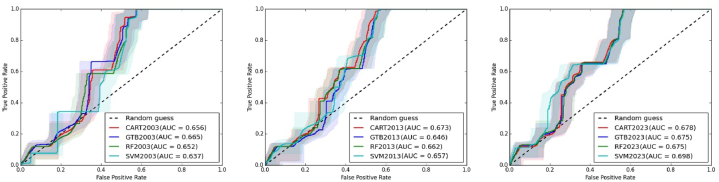
AUC curves of CART, GTB, RF, and SVM algorithms.

Table 7 and Fig. 9 represent the ROC curve of CART, GTB, RF, and SVM algorithms and AUC values. To calculate the AUC, 10,000 representative values were used. In the AUC value, we can see that only two AUC values (SVM 2003 and GTB 2023) are below 0.65. An AUC value above 0.6 considers satisfactory classification accuracy. Here all the AUC values are above the threshold.

**Table 7:** AUC values for different ML algorithms.

	CART	GTB	RF	SVM
2003	0.656	0.665	0.652	0.637
2013	0.678	0.675	0.675	0.698
2023	0.673	0.646	0.662	0.657

### 4.2 LULC dynamics and magnitudes

**Fig. 10:**
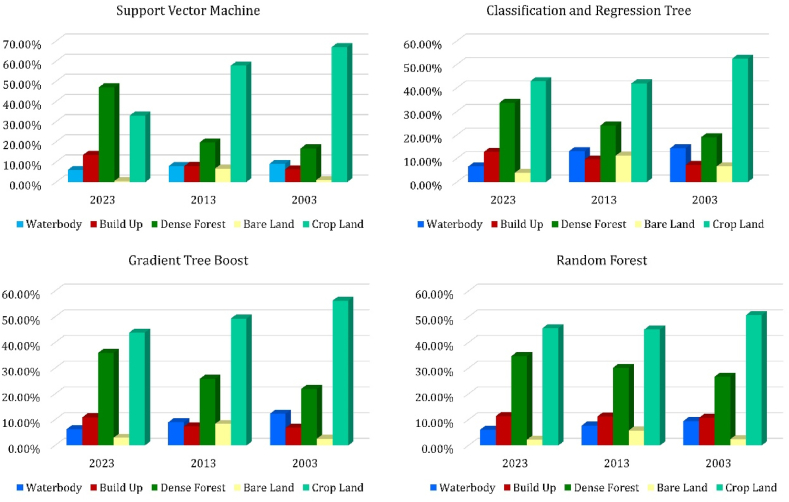
Percentage of LULC class of the total area in Chattogram.

**Fig. 11:**
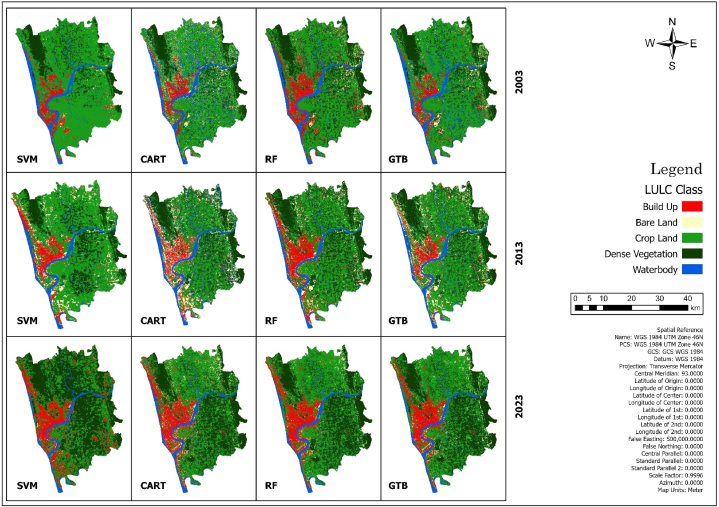
Multi-temporal LULC maps of Chattogram metropolitan area.

In this study, we used four machine learning algorithms to classify land use land cover for the study area (Fig. 11). Fig. 10 shows the percentages of LULC class of the total area for the four machine learning algorithms used in this study.

The SVM algorithm has classified a gradual decrease in the waterbody area in the Chattogram Metropolitan Area. In 2003, the waterbody accounted for 9.08 % of the total area, which decreased to 7.91 % in 2013 and 5.99 % in 2023. The SVM algorithm also indicates a sharp decrease in cropland. In 2003 and 2013, cropland comprised the highest percentage of the total area, with 66.97 % and 57.76 %, respectively. However, by 2023, it decreased to 33.02 %.

On the other hand, bare land had the lowest percentage in 2003 and 2023, accounting for 0.93 % and 0.49 %, respectively. It increased to 6.72 % in 2013 before declining. The built-up area expanded from 6.33 % in 2003 to 13.52 % in 2023. According to the SVM algorithm, dense vegetation has significantly increased, with a 30.29 % rise from 2003 to 2023 in the Chattogram Metropolitan Area.

The total study area is 1163.60 square kilometers, and the CART algorithm identified 167.50 sq. km (14.39 %) of the area covered with waterbodies in 2003. From 2003 to 2013, as per CART classification, the waterbody area decreased by 1.20 %, and during 2013–2023, it decreased by 6.54 %, which is 5.43 times higher than 2003–2013. The cropland comprises the highest percentage of land cover in all three years. Nevertheless, between 2003 and 2013, 10.60 % of cropland was converted into other land use, and overall, 9.61 % decrease from 2003 to 2023.

The build-up area takes up 7.30 %, 9.60 %, and 12.82 % of land in 2003, 2013, and 2023 respectively. So, there is a total of 5.52 % of the increase in build-up area from 2003 to 2023. The dense vegetation increases from 19.10 % to 33.67 % in these 20 years. The bare land transition is not smooth like other land use. 2003, it covered 6.71 % of the total land and increased by 4.57 % in 2013. Then in 2023, it decreases by 7.31 % and takes only 3.96 % of the total area.

The GTB and CART algorithms perform nearly the same. GTB classifies 12.33 % of the waterbody in 2003, 9.05 % in 2013, and 6.35 % in 2023. From 2003 to 2023, about 6 % of waterbody converted into other land use. As per GTB, there was 654.09 sq. km of cropland in 2003, but in 20 years, cropland was reduced by about 145 sq km.

The dense vegetation increased by about 3.98 % between 2003 and 2013 and another 9.65 % in the next ten years (2013–2023). There is a gradual increase in the build-up area also. Between 2003 and 2013, there was a slight change in the build-up area. As per GTB, only 6.66 sq km of new built-up area have been built in these ten years. In the next ten years (2013–2023), there is an increase of 3.48 % in the build-up area. As usual in bare land, it increased from 2003 to 2013 and decreased from 2013 to 2023.

The Random Forest algorithm classifies the build-up area relatively close to each year. There is only a 0.63 % increase in build-up area in 20 years. It identifies 10.74 % of the build-up area in 2003, 11.24 % in 2013, and 11.37 % in 2023. The increase in dense vegetation is also relatively lower than other algorithms. According to the CART and GTB classifier, there is more than a 10 % change in dense vegetation. In contrast, RF identifies an 8 % change in dense vegetation in these 20 years. As per RF classifier, about 50.61 % of the total land was cropland in 2003, which decreased by 5.58 % between 2003 and 2013.

There is little or no change in cropland between 2013 and 2023. In 2003, about 9.50 % of the total land was waterbodies; in 2013, it became 7.74 %. Overall, the waterbody decreases by 3.38 % during 2003–2023. The bare land follows the same trend as other algorithms. In 2003 there was 2.43 % of bare land, which increased to 5.83 % in 2013 and then reduced to 2.24 % in 2023.

### 4.3 LULC conversion matrix

**Fig. 12:**
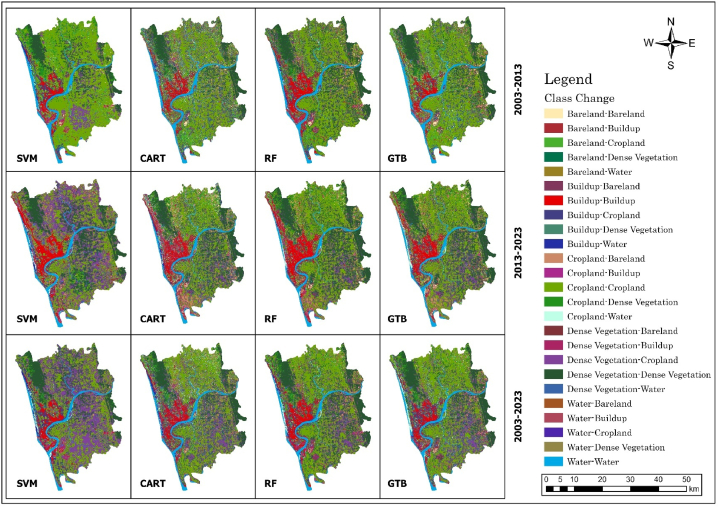
Spatial transformation in LULC class during 2003–2013, 2013–2023, and 2003–2023.

The spatial LULC, change or conversion matrix, has been carried out among three different periods, i.e., 2003–2013, 2013–2023, and 2003–2023 (Table 9 and Fig. 12). LULC conversion has been illustrated in three ways.

i. Particular LULC class to specific LUCL class (No Change)
ii. Transformation of certain classes to other LULC classes
iii. Transformation of other LULC classes to specific LULC class

**Table 8:** Statistics of LULC from 2003 to 2023 at 10-years intervals in Chattogram city.

Algorithm	Year	Build Up		Bare Land		Cropland		Dense Vegetation		Waterbody	
		Area (sq. km)	Area (%)	Area (sq. km)	Area (%)	Area (sq. km)	Area (%)	Area (sq. km)	Area (%)	Area (sq. km)	Area (%)
SVM	2003	73.65	6.33 %	10.81	0.93 %	779.25	66.97 %	194.25	16.69 %	105.64	9.08 %
	2013	92.39	7.94 %	78.23	6.72 %	672.11	57.76 %	228.79	19.66 %	92.08	7.91 %
	2023	157.35	13.52 %	5.69	0.49 %	384.17	33.02 %	546.70	46.98 %	69.70	5.99 %
CART	2003	84.88	7.30 %	78.10	6.71 %	610.91	52.50 %	222.21	19.10 %	167.50	14.39 %
	2013	111.65	9.60 %	131.23	11.28 %	487.61	41.90 %	279.52	24.02 %	153.58	13.20 %
	2023	149.16	12.82 %	46.12	3.96 %	499.05	42.89 %	391.79	33.67 %	77.48	6.66 %
GTB	2003	79.62	6.84 %	30.84	2.65 %	654.09	56.21 %	255.62	21.97 %	143.43	12.33 %
	2013	86.28	7.41 %	97.73	8.40 %	573.54	49.29 %	300.80	25.85 %	105.26	9.05 %
	2023	126.74	10.89 %	34.80	2.99 %	510.18	43.84 %	418.01	35.92 %	73.87	6.35 %
RF	2003	124.93	10.74 %	28.31	2.43 %	588.89	50.61 %	310.97	26.72 %	110.50	9.50 %
	2013	130.73	11.24 %	67.88	5.83 %	523.93	45.03 %	350.95	30.16 %	90.10	7.74 %
	2023	132.31	11.37 %	26.12	2.24 %	530.31	45.57 %	403.72	34.70 %	71.14	6.11 %

**Table 9:** Statistics of periodical change between different time points in Chattogram city.

Algorithm	Year	Build Up		Bare Land		Cropland		Dense Vegetation		Waterbody	
		Area (sq. km)	Area (%)	Area (sq. km)	Area (%)	Area (sq. km)	Area (%)	Area (sq. km)	Area (%)	Area (sq. km)	Area (%)
SVM	2003–2013	18.74	1.61 %	67.42	5.79 %	−107.14	−9.21 %	34.54	2.97 %	−13.56	−1.17 %
	2013–2023	64.96	5.58 %	−72.54	−6.23 %	−287.94	−24.75 %	317.91	27.32 %	−22.38	−1.92 %
	2003–2023	83.70	7.19 %	−5.12	−0.44 %	−395.08	−33.95 %	352.45	30.29 %	−35.94	−3.09 %
CART	2003–2013	26.77	2.30 %	53.13	4.57 %	−123.30	−10.60 %	57.31	4.93 %	−13.92	−1.20 %
	2013–2023	37.51	3.22 %	−85.12	−7.31 %	11.45	0.98 %	112.27	9.65 %	−76.10	−6.54 %
	2003–2023	64.28	5.52 %	−31.98	−2.75 %	−111.85	−9.61 %	169.58	14.57 %	−90.02	−7.74 %
GTB	2003–2013	6.65	0.57 %	66.89	5.75 %	−80.55	−6.92 %	45.18	3.88 %	−38.18	−3.28 %
	2013–2023	40.46	3.48 %	−62.93	−5.41 %	−63.35	−5.44 %	117.21	10.07 %	−31.39	−2.70 %
	2003–2023	47.12	4.05 %	3.96	0.34 %	−143.91	−12.37 %	162.40	13.96 %	−69.56	−5.98 %
RF	2003–2013	5.80	0.50 %	39.58	3.40 %	−64.95	−5.58 %	39.98	3.44 %	−20.40	−1.75 %
	2013–2023	1.58	0.14 %	−41.77	−3.59 %	6.38	0.55 %	52.77	4.53 %	−18.96	−1.63 %
	2003–2023	7.38	0.63 %	−2.19	−0.19 %	−58.57	−5.03 %	92.75	7.97 %	−39.36	−3.38 %

These have been illustrated together for each distinct representation of LULC class, i.e., build-up, bare land, cropland, dense vegetation, and waterbody for each machine learning algorithm.

**Fig. 13:**
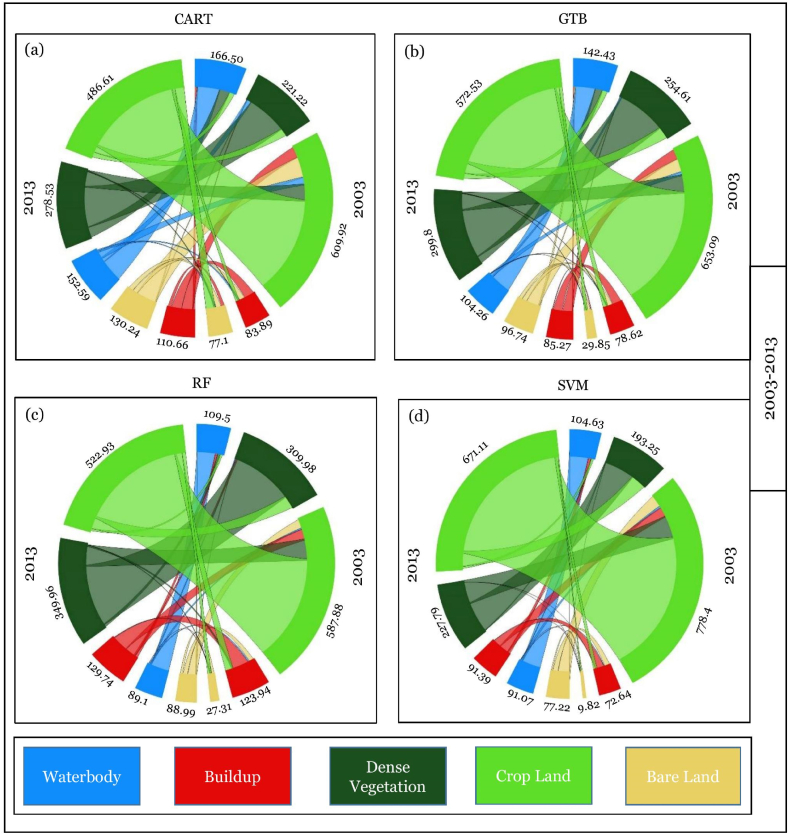
Chord diagram of LULC conversion from 2003 to 2013.

The changing pattern of the LULC was displayed using a chord diagram. Fig. 13 represents the changes from 2003 to 2013, 2013–2023, and 2003–2023. The LULC conversion matrix for the last 20 years in Chattogram Metropolitan Area discovered that the “build up” and “dense vegetation” areas have increased, whereas the “cropland” and “waterbody” areas have decreased (Table 10.

**Table 10:** LULC transformation from 2003 to 2013.

		*Waterbody*	*Build Up*	*Dense Vegetation*	*Bare Land*	*Cropland*	Total
CART	*Waterbody*	89.42	9.32	42.59	4.79	21.38	167.50
	*Build Up*	6.77	41.07	1.93	21.10	14.02	84.88
	*Dense Vegetation*	20.72	3.30	162.94	3.02	32.24	222.21
	*Bare Land*	4.76	12.75	4.25	19.69	36.65	78.10
	*Cropland*	31.92	45.22	67.82	82.64	383.32	610.91
	**Total**	153.58	111.65	279.52	131.23	487.61	1163.60
GTB	*Waterbody*	78.80	4.75	30.45	7.62	21.81	143.43
	*Build Up*	2.31	36.26	2.63	19.41	19.01	79.62
	*Dense Vegetation*	7.71	2.11	193.75	3.57	48.47	255.62
	*Bare Land*	0.38	2.79	1.58	13.85	12.25	30.84
	*Cropland*	16.06	40.36	72.39	53.29	471.99	654.09
	**Total**	105.26	86.28	300.80	97.73	573.54	1163.60
RF	*Waterbody*	73.46	10.55	12.80	3.31	10.38	110.50
	*Build Up*	4.59	65.35	6.65	17.76	30.59	124.93
	*Dense Vegetation*	4.69	7.88	244.93	1.41	52.07	310.97
	*Bare Land*	0.36	2.28	1.84	9.83	14.00	28.31
	*Cropland*	7.00	44.68	84.74	35.57	416.89	588.89
	**Total**	90.10	130.73	350.95	67.88	523.93	1163.60
SVM	*Waterbody*	78.64	12.79	3.29	0.98	9.93	105.64
	*Build Up*	1.74	35.96	0.36	21.43	14.15	73.65
	*Dense Vegetation*	1.91	0.26	128.12	0.72	63.24	194.25
	*Bare Land*	0.12	0.51	0.19	5.69	4.31	10.81
	*Cropland*	9.66	42.87	96.83	49.40	580.48	779.25
	**Total**	92.08	92.39	228.79	78.23	672.11	1163.60

#### 4.3.1 LULC conversion from 2003 to 2013

Table 10 shows the LULC change from 2003 to 2013. Table 9 and Fig. 13(a–d) depict LULC conversion from 2003 to 2013. The LULC change is stated in terms of square kilometers. Most changes in the classes category between 2003 and 2013 were negative values except for built-up and dense vegetation classes. The CART algorithm identifies (Fig. 13a) that 9.32 sq km of waterbody, 3.30 sq km of dense vegetation, 12.75 sq km of bare land, and 45.22 sq km of cropland converted into the built-up area between 2003 and 2013. As cropland is the dominant class among these five classes, it transforms most into other classes. From cropland class, 31.92 sq km transformed into a waterbody, 67.82 sq km into dense vegetation, and 82.64 sq km into bare land. There was also a sharp decrease in the waterbody area from 2003 to 2013, and about 42.59 sq km of waterbody turned into dense vegetation and 21.38 sq km of land converted into cropland LULC class.

On the other hand, according to the GTB classifier (Fig. 11b), from 2003 to 2013, 4.75 sq km of waterbody, 2.11 sq km of dense vegetation, 2.79 sq km of bare land, and 40.36 sq km of cropland converted into built-up area. This classifier also identifies an increase in dense vegetation. The cropland (72.39 sq km) and waterbody (30.45 sq km) are mostly converted into dense vegetation. Furthermore, 30.45 sq km and 21.81 sq km of waterbody transformed into dense vegetation and cropland, respectively.

Whereas the RF classifier identifies (Fig. 13c) 44.68 sq km of cropland, 10.55 sq km of waterbody, 7.88 sq km of dense vegetation, and 2.28 sq km of bare land conversion into built-up area. There was minimal conversion between other classes to the waterbody. The most considerable amount of land converted into waterbody was from cropland (7.00 sq km). The conversion matrix also shows that 35.57 sq km of cropland was converted into bare land from 2003 to 2013. RF algorithm measures that 52.07 sq km of dense vegetation converted into cropland vis-e-vis 84.74 sq km transformed from cropland to dense vegetation.

Finally, through the SVM classifier, between 2003 and 2013, waterbody areas were mainly converted into build-up and cropland areas, respectively 12.79 sq km and 9.93 sq km (Fig. 13d). The highest amount of land conversion occurred between cropland to dense vegetation (96.83 sq km) vis-e-vis 63.24 sq km of dense vegetation also transformed into cropland from 2003 to 2013 as per the SVM algorithm. The enormous amount of land transformed into built-up is from cropland class (42.87 sq km). Dense vegetation and bare land converted 0.26 sq km and 0.51 sq km into the built-up area.

In summary, the waterbody and cropland reduced significantly in these ten years, whereas there was a sharp increase in built-up, bare land, and dense vegetation. The most dominated LULC class was the cropland and the most converted one (SVM = 198.77 sq km; CART = 227.59 sq km; GTB = 182.09 sq km; and RF = 172.00 sq km). The next most converted one is waterbody, as per SVM, CART, GTB, and RF, 27.00 sq km, 78.08 sq km, 64.43 sq km, and 37.05 sq km, respectively, transformed into other land use. Among these four algorithms, the conversion of dense vegetation is relatively constant; the SVM and RF show there was 66 sq km of dense vegetation converted into other LULC classes and 59.27 sq km and 61.86 sq km, respectively, by CART and GTB.

#### 4.3.2 LULC conversion from 2013 to 2023

**Fig. 14:**
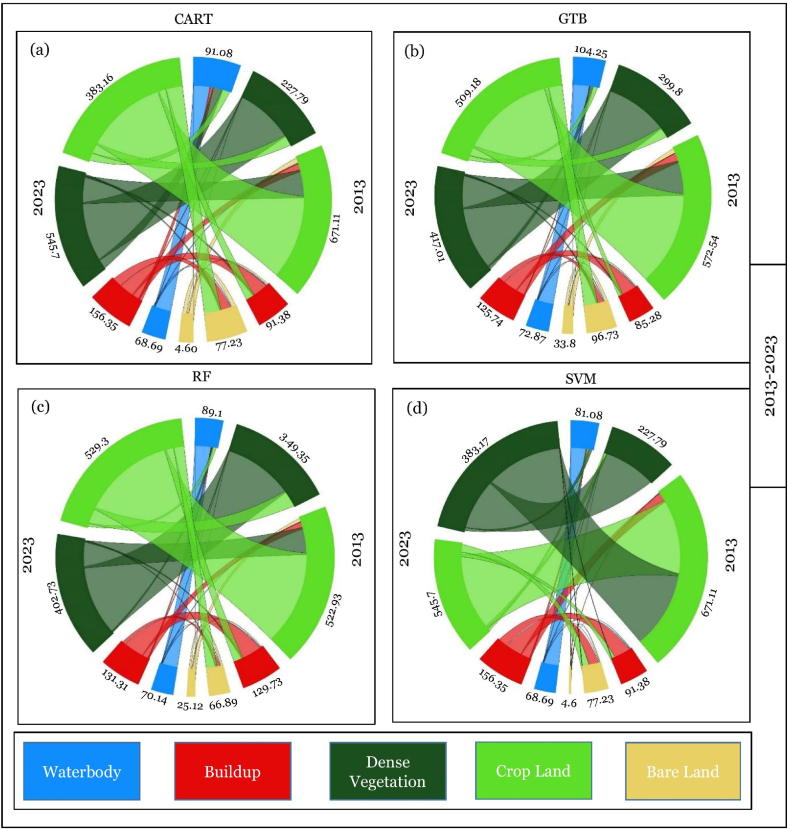
Chord diagram of LULC conversion from 2013 to 2023.

Table 8 shows the LULC change from 2003 to 2023. Table 11 and Fig. 14(a–d) illustrate the LULC-classes transformation from 2013 to 2023. Three classes' category changes were negative as per SVM and GTB; for CART and RF, it was two, which means loss of waterbody, bare land, and cropland.

**Table 11:** LULC transformation from 2013 to 2023.

		*Waterbody*	*Build Up*	*Dense Vegetation*	*Bare Land*	*Cropland*	Total
CART	*Waterbody*	66.71	14.22	40.16	1.28	31.22	153.58
	*Build Up*	2.69	60.98	7.37	6.23	34.40	111.65
	*Dense Vegetation*	1.93	2.76	231.05	0.79	43.00	279.52
	*Bare Land*	2.08	39.41	6.45	16.68	66.62	131.23
	*Cropland*	4.08	31.81	106.75	21.15	323.82	487.61
	**Total**	77.48	149.16	391.79	46.12	499.05	1163.60
GTB	*Waterbody*	66.47	3.57	15.34	0.58	19.29	105.26
	*Build Up*	1.55	60.13	4.46	2.66	17.48	86.28
	*Dense Vegetation*	0.82	1.80	254.67	0.81	42.70	300.80
	*Bare Land*	2.57	22.07	6.67	11.52	54.90	97.73
	*Cropland*	2.46	39.17	136.87	19.23	375.81	573.54
	**Total**	73.87	126.74	418.01	34.80	510.18	1163.60
	*Waterbody*	63.88	4.58	7.66	0.30	13.68	90.10
RF	*Build Up*	2.44	82.15	6.10	3.30	36.74	130.73
	*Dense Vegetation*	0.86	2.76	279.63	0.71	66.99	350.95
	*Bare Land*	1.08	14.67	2.64	7.75	41.75	67.88
	*Cropland*	2.88	28.15	107.70	14.06	371.14	523.93
	**Total**	71.14	132.31	403.72	26.12	530.31	1163.60
SVM	*Waterbody*	62.11	6.66	13.73	0.22	9.36	92.08
	*Build Up*	2.70	67.81	2.33	1.04	18.50	92.39
	*Dense Vegetation*	0.85	1.10	212.78	0.03	14.03	228.79
	*Bare Land*	0.46	40.69	2.58	2.66	31.84	78.23
	*Cropland*	3.57	41.09	315.28	1.74	310.43	672.11
	**Total**	69.70	157.35	546.70	5.69	384.17	1163.60

So, for the CART classifier (Figs. 14a), 14.22 sq km waterbody, 2.76 sq km dense forest, 39.41 sq km bare land, and 31.81 sq km cropland were converted into the built-up area from 2013 to 2023. The waterbody became half between 2013 and 2023. Most of the waterbody converted into dense vegetation (40.16 sq km) and cropland (31.22 sq km). The most transformation occurred in bare land. In 2013, there was 131.23 sq km of bare land. However, in 2023, it reduced to 46.12 sq km. Bare land is mostly converted into built-up and cropland. 86.87 sq km of the waterbody, 48.47 sq km of dense vegetation, 114.56 sq km of bare land, and 163.79 sq km of cropland converted into different classes in these ten years.

As per the GTB classifier (Fig. 14b), there was 86.28 sq km of built-up area in 2013, which increased to 126.74 sq km in 2023. Cropland mainly transformed into a built-up area from 2013 to 2023, about 39.17 sq km, whereas 22.07 sq km of bare land, 3.57 sq km waterbody, and 1.80 sq km of dense forest also transformed into a built-up area. Furthermore, there is also a decrease of 86.21 sq km of bare land and 197.73 sq km of cropland into other classes. The most increased LULC is the dense vegetation, which increased from 300.80 sq km to 418.01 sq km during these periods.

On the other hand, the RF algorithm identifies (Fig. 14c) that there was a decrease in waterbody and bare land from 2013 to 2023. There is a slight increase in cropland and built-up area from 523.93 sq km to 530.21 sq km and 130.73 sq km to 132.31 sq km, respectively, from 2013 to 2023. The highest increase occurs in the dense vegetation class, with about 107.70 sq km of cropland converted to dense vegetation, and overall, it skyrocketed from 350.95 sq km to 403.72 sq km. The bare land decreased from 67.88 sq km to 26.12 sq km, and approximately 14.67 sq km of bare land transformed into a built-up area. The cropland contributed 14.67 sq km in these ten years, the highest amount of land converted into the built-up class.

Finally, the SVM algorithm measures (Fig. 14d) a massive decrease in cropland and a massive increase in dense vegetation. About half of the cropland is converted into dense vegetation per the SVM classifier. There were 672.11 sq km of cropland in 2023, and it reduced to 384.17 sq km in 2023, where 315.28 sq km of cropland transformed into dense vegetation. There is also a sharp increase in built-up area, which rose from 92.39 sq km to 157.35 sq km. Bare land and cropland contribute almost equally to this increment, 40.69 sq km, and 41.09 sq km, respectively. On the other hand, 6.66 sq km of waterbody and 1.10 sq km of dense vegetation were also converted into built-up areas from 2013 to 2023.

The CART algorithm measures the most change of 86.87 sq km of waterbody transform into other classes, where GTB, RF, and SVM identify 38.79 sq km, 26.22 sq km, and 29.96 sq km, respectively. Much change is occurring in the bare land class, and it increases between 2003 and 2013. However, it will decrease a lot in the next ten years. As per CART, GTB, RF, and SVM, 114.56 sq km, 86.21 sq km, 60.14 sq km, and 75.57 sq km, respectively converted into other classes. The most surprising result was the SVM classifier classification results for 2023. The dense vegetation jumped from 228.79 sq km to 546.70 sq km and a considerable decrease in cropland which dropped from 672.11 sq km to 384.17 sq km.

#### 4.3.3 LULC conversion from 2003 to 2023

**Fig. 15:**
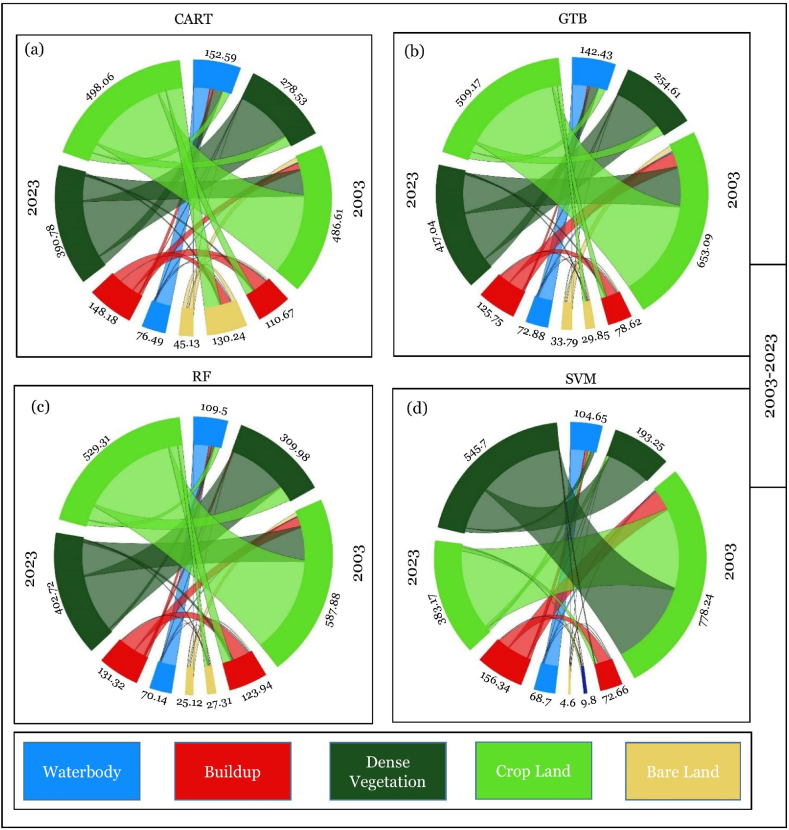
Chord diagram of LULC conversion from 2003 to 2023.

Table 12 and Fig. 15(a–d) depict the LULC transformation from 2003 to 2023. For all classifiers, built-up and waterbody areas experienced the most remarkable changes between 2003 and 2023.

**Table 12:** LULC transformation from 2003 to 2023.

		*Waterbody*	*Build Up*	*Dense Vegetation*	*Bare Land*	*Cropland*	Total
CART	*Waterbody*	62.55	20.58	50.00	1.39	32.97	167.50
	*Build Up*	3.98	49.23	5.06	4.57	22.05	84.88
	*Dense Vegetation*	1.56	5.70	177.22	1.48	36.25	222.21
	*Bare Land*	2.28	16.36	11.33	6.45	41.68	78.10
	*Cropland*	7.11	57.30	148.18	32.23	366.09	610.91
	**Total**	77.48	149.16	391.79	46.12	499.05	1163.60
GTB	*Waterbody*	63.13	9.21	39.31	1.08	30.70	143.43
	*Build Up*	2.35	43.99	6.68	4.42	22.18	79.62
	*Dense Vegetation*	1.21	3.66	204.28	1.23	45.23	255.62
	*Bare Land*	0.35	4.81	4.86	4.12	16.71	30.84
	*Cropland*	6.84	65.08	162.88	23.94	395.35	654.09
	**Total**	73.87	126.74	418.01	34.80	510.18	1163.60
RF	*Waterbody*	62.53	11.36	13.57	0.74	22.30	110.50
	*Build Up*	3.40	65.59	12.08	5.46	38.41	124.93
	*Dense Vegetation*	1.03	8.02	234.51	0.81	66.61	310.97
	*Bare Land*	0.35	2.95	3.75	2.76	18.50	28.31
	*Cropland*	3.83	44.40	139.81	16.35	384.49	588.89
	**Total**	71.14	132.31	403.72	26.12	530.31	1163.60
SVM	*Waterbody*	59.83	15.54	17.36	0.64	12.28	105.64
	*Build Up*	2.20	48.70	3.83	2.27	16.66	73.65
	*Dense Vegetation*	0.75	0.93	177.67	0.03	14.87	194.25
	*Bare Land*	0.13	3.24	0.95	0.46	6.02	10.81
	*Cropland*	6.79	88.93	346.89	2.29	334.34	779.25
	**Total**	69.70	157.35	546.70	5.69	384.17	1163.60

The RF classifier (Fig. 15c) identifies the minimum change in built-up areas among these four machine learning algorithms. In 2003, there were 124.93 sq km of built-up areas, which increased to 132.31 sq km in 2023. As per this classifier, only the waterbody class reduced a lot from 110.50 sq km to 71.14 sq km in these 20 years.

As per the CART algorithm (Fig. 15a), the waterbody, bare land, and cropland were significantly reduced. On the other hand, the built-up area and dense vegetation skyrocketed between 2003 and 2023. The waterbody reduced from 167.50 sq km to 77.48 sq km. The cropland was also deducted from 610.91 sq km to 499.05 sq km. Between 2003 and 2023, 20.58 sq km of waterbody, 5.70 sq km of dense vegetation, 16.36 sq km of bare land, and 57.30 sq km of cropland turned into a built-up area. In these 20 years, 148.18 sq km of cropland was converted to dense vegetation. Most of the waterbody area (50 sq km) turned into dense vegetation.

For the GTB classifier (Fig. 15b), there was 143.43 sq km of waterbody in 2003, which was reduced to 73.84 sq km in 2023. The cropland and the bare land also follow the decreasing trend, whereas the built-up and dense vegetation follows the increasing trend. The built-up area rose from 79.62 sq km to 126.74 sq km. In these 20 years, 9.21 sq km of waterbody, 3.66 sq km of dense forest, 4.81 sq km of bare land, and 65.08 sq km of cropland turned into built-up areas. The cropland was reduced by about 144 sq km in these 20 years.

Finally, the SVM classifier (Fig. 15d) classifies that there was a boom in dense vegetation. In 2003 there was only 194.24 sq km of dense vegetation. However, in 2023 increased to 546.70 sq km. Subsequently, the cropland was reduced a lot, it was 779.25 sq km in 2003, and it deducted to 384.17 sq km in 2023. The built-up areas also skyrocketed in these 20 years, increasing from 73.65 sq km to 157.35 sq km.

### 4.4 Comparison among different classifiers

This study used four machine learning algorithms for LULC classification under the same condition. Every machine learning algorithm's efficiency and performance depend on certain conditions [50]. The SVM classifier performs relatively poorer in the accuracy assessment than the other three algorithms (GTB, CART, and RF). The accuracy of RF and CART classifiers is relatively the same over the three time periods, where the accuracy of GTB is comparatively lower in 2003 than in the other two periods (2013 and 2023). In this study, the CART and GTB algorithms performed similarly. The CART algorithm has estimated built-up areas from 7.30 % to 12.82 %, whereas the GTB has estimated 6.84 %–10.89 %. There are small fractions in LULC classes classified by CART and GTB algorithms. The RF classifier overestimated the built-up areas in 2003 and 2013, whereas the SVM overestimated the dense vegetation and underestimated the cropland in 2023. All four algorithms identify the cropland similarly, except for the SVM classifier in 2023. The waterbody is classified more or less the same by all four types of algorithms. All the algorithms identify an increase in dense vegetation in the CMA. Also, all the algorithms identified an increase in bare land area from 2003 to 2013 and then a decrease from 2013 to 2023. So, in this study, the conditions are favorable for CART and GTB algorithms which is why these algorithms perform relatively well than RF and SVM classifiers.

### 4.5 Optimal classifier for urban sprawl

In the previous section, this study discussed the results of LULC change and compared these four machine learning algorithms. Based on the result the study found that the CART model is faster than GTB and RF as those models use iteration process which required more time and power. The SVM classifier did not show the promising result in this study area context. Based on the results accuracy, this study found the classification and regression tree (CART) algorithm is the most suitable for identifying the urban sprawl trend from 2003 to 2023. The overall accuracy of the model with the limited data is preferable.

The CART classifier identifies that the urban area gradually increased from 10.74 % to 11.37 % of the total areas. The anomaly in bare land found in the analysis due to the different mega construction projects took place in that period in Chattogram especially the Karnaphuli Economic Zone. From 2013 to 2023, about 39 square kilometers of bare land converted into built up area. As the construction of those mega projects finished before 2013 that could be a possible reason why the bare land area again decreased in 2023. Due to rapid urbanization the study area lost approximately 10 % of its agricultural land in the last 20 years. Aside from agricultural land, the study region has a diminishing trend for its waterbody, which has declined 7.74 % over a 20-year period. The CART classifier identified the trend of increasing 14.57 % of dense vegetation in the study area. From an environmental and ecological perspective, it is a good sign. However, by further investigation, it was found that due to the 30-m spatial resolution, the algorithm straggle to identify the urban sprawl in the distant fringe area where the big trees are situated near small buildings and the classifier classified those areas as dense vegetation. So, from this study area perspective, it is a sign of uncontrolled urban expansion.

Mapping the LULC dynamics from time to time can help to monitor the urban sprawl and act accordingly. Remote sensing and CART algorithms can come very handy for this study area for this continuous investigation. However, there are some drawbacks like moderate spatial resolution imagery, selecting satellite imagery from different time periods. Limited budget and time, and access to large quantities of high-resolution imageries and computational resources for training more advanced algorithms like deep learning are few limitations of this study.

To summarize, machine learning algorithm CART is optimal algorithm for this study for land use land cover classification due to its established efficacy, interpretability, and efficiency.

### 4.6 Importance of LULC monitoring in urban sustainability

**Fig. 16:**
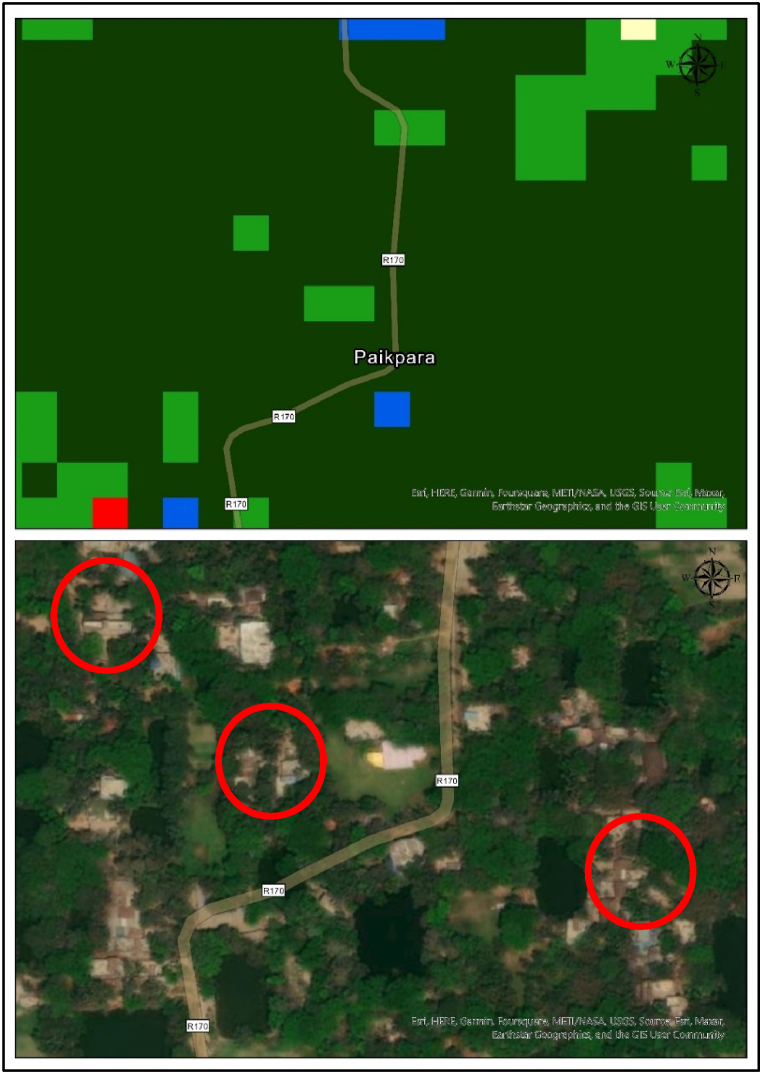
Validation using ESRI firefly image.

The increase in the built-up area has come at the expense of other LULC classes due to the quick conversion of LULC classes. From the above discussion, we have seen that all four algorithms identify the shrunk of cropland and waterbody, paving the way for the expansion of the built-up area. The dense vegetation has dramatically increased in the Chattogram Metropolitan Area in these 20 years. However, we have used 30 m Landsat image for the classification, and it is hard to identify the built-up area near the dense vegetation. Let us closely examine the ESRI Firefly Image (Fig. 16). We can see that in the rural part of the study area, built-up grew under the dense vegetation since there is a trend among Bangladesh's rural people that they plant big trees surrounding their households to avoid the extreme heat.

However, one of the drawbacks of using 30 m resolution image failed to identify those built-up areas covered with large trees. On the contrary, in the urban part of the study area, the other LULC classes were converted into built-up areas at a rapid speed. This pattern suggests sudden shifts that severely impact the systems and processes of urban ecology and the population's access to food [[51], [52], [53]]. So, by determining where and how the dynamics of the land have changed, LULC mapping and monitoring is currently one of the effective measures in minimizing the vulnerability of urban sustainability. It allows for the spatial measurement of trends, patterns, and magnitudes. This knowledge development can help combat the issues at hand, whether connected to food security and access, unplanned urban sprawl, or the advantages of dense vegetation. The spatial data of various LULC classes and their transformation can provide a proper understanding of “where, what, and how” has occurred on the city landscape, which can help to form effective planning and policy for enhancing the carrying capacity of the Chattogram Metropolitan Area as well as its sustainability.

Urban ecologists, policymakers, planners, and environmental experts concentrated on minimizing the adverse effects of LULC alteration in climate change on a local to global scale. Assessing the LULC change on a local to global scale can help identify the effects of climate change, such as global warming, deforestation, droughts, and agricultural stress [54,55]. To remove the main barriers to urban growth, the CDA (Chattogram Development Authority) created a master plan for the Chattogram Metropolitan Area in 1996. The Chattogram Metropolitan Master Plan (2020–2041) is now being revised by the CDA, focusing on the urban structure plan, drainage master plan, transportation master plan, and environment management plan. Therefore, this study based LULC assessment and estimation of the cityscape change can help minimize any unintended repercussions of climate change. The Chattogram Metropolitan Area's unchecked urban sprawl growth can be reduced by smart urban planning, another benefit of this study.

## 5 Conclusion

This study utilized Google Earth Engine and GIS to analyze and quantify land use and land cover changes in the Chattogram Metropolitan Area over 20 years from 2003 to 2023. Four machine learning algorithms (CART, GTB, RF, and SVM) were integrated to assess these changes. The study also employed statistical analysis and chord diagrams to visualize the land use and land cover transformations between 2003, 2013, and 2023, focusing on five identified classes.

The classification accuracy of the four machine learning algorithms was evaluated using the confusion matrix and receiver operating characteristic (ROC) curve. The overall accuracy of the classification was deemed satisfactory based on these assessments. The findings revealed significant land use and land cover transformations in the Chattogram Metropolitan Area during the study period. The built-up areas exhibited a consistent annual growth trend, ranging from 0.42 % to 4.80 % across the different algorithms. In contrast, waterbodies experienced a declining trend, with an annual decrease ranging from 2.06 % to 5.16 %. Conversely, cropland significantly decreased over the 20 years, with an average yearly decrease ranging from 3.36 % to 22.64 %. However, the study noted limitations in capturing built-up areas located in close proximity to dense vegetation due to the resolution limitations of the 30-m imagery used.

This analysis provides valuable information for planners and policymakers to understand the yearly trends in land use and land cover dynamics, enabling sustainable land management planning. The expansion of built-up areas was observed at the expense of croplands and waterbodies, highlighting the need for ongoing monitoring of land use and land cover conversions to ensure long-term environmental sustainability. The study suggested utilizing archives of Landsat 5 (TM), Landsat 8 (OLI), and Landsat 9 (OLI-2) imagery, as well as newly developed remote sensing satellite data, to create accurate maps and monitor future land use and land cover transformations in the Chattogram Metropolitan Area.

## Data availability

Data will be made available on request.

## Additional information

No additional information is available for this paper.

## CRediT authorship contribution statement

**Jayanta Biswas:** Conceptualization, Formal analysis, Methodology, Writing – original draft, Writing – review & editing, Visualization. **Md Abu Jobaer:** Formal analysis, Visualization, Writing – original draft, Writing – review & editing. **Salman F. Haque:** Data curation, Writing – original draft, Writing – review & editing. **Md Samiul Islam Shozib:** Data curation, Writing – original draft, Writing – review & editing. **Zamil Ahamed Limon:** Formal analysis, Writing – original draft, Writing – review & editing.

## Declaration of competing interest

The authors declare that they have no known competing financial interests or personal relationships that could have appeared to influence the work reported in this paper.
